# Isolation and Identification of Algicidal Compound from *Streptomyces* and Algicidal Mechanism to *Microcystis aeruginosa*

**DOI:** 10.1371/journal.pone.0076444

**Published:** 2013-10-03

**Authors:** Jianfei Luo, Yuan Wang, Shuishui Tang, Jianwen Liang, Weitie Lin, Lixin Luo

**Affiliations:** College of Bioscience and Bioengineering, South China University of Technology, Guangzhou, Pr China; University of New South Wales, Australia

## Abstract

The biological control of cyanobacterial harmful algal blooms (cyanoHABs) is important to promote human health, environmental protection, and economic growth. Active algicidal compounds and algicidal mechanisms should be identified and investigated to control cyanoHABs. In this study, the algicidal actinobacterium *Streptomyces* sp. L74 was isolated from the soil of a nearby pond which located in the center lake of Guanghzou Higher Education Mega Center. Results showed that the algicidal activities of cyanoHABs are mainly achieved via an indirect attack by producing algicidal compounds. All active algicidal compounds are hydrophilic substances that are heat and pH stable. In the present study, an active compound (B3) was isolated and purified by high-performance liquid chromatography and identified as a type of triterpenoid saponin (2-hydroxy-12-oleanene-3, 28-O-D-glucopyranosyl) with a molecular formula of C_42_H_70_O_13_ as determined by infrared spectrometry, electrospray ionization mass spectrometry, and nuclear magnetic resonance. Active algicidal compounds from *Streptomyces* sp. L74 were shown to disrupt the antioxidant systems of *Microcystis aeruginosa* cells.

## Introduction

The growth of cyanobacterial harmful algal blooms (cyanoHABs) has become a global concern as they threaten the environment, economy, and human health and require treatment to control pollution. Chemical, physical, and biological treatments have been applied to control cyanoHABs in aquatic environments [1–3]. However, chemical and physical methods entail high costs and cause secondary pollution; hence, rapid and highly active biological methods are considered as important tools to control cyanoHABs [4].

In nature, cyanoHABs are biologically controlled by microorganisms exhibiting algicidal activities. These microorganisms kill cyanobacteria by attacking the cells either directly via cell-to-cell contact or indirectly via the release of algicidal compounds [5,6]. *Vibrio*, *Pseudoalteromonas*, *Bacillus*, *Pseudomonas*, *Ateromonas*, and *Micrococcus* spp. are common algicidal microorganisms [3,6,7]. These bacteria secrete algicidal substances, including proteins, peptides, amino acids, antibiotics, nitrogenous compounds, and alkaloids [8–14]. However, few algicidal compounds have been isolated and purified. Furthermore, algicidal mechanisms, which may elucidate the variations in characteristics among different species of algicidal bacteria, are seldom determined [15].

Previous studies have shown that bacteria, viruses, fungi, and actinobacteria exhibit algicidal activities [16–18]. However, the high specificity of viruses to hosts and the parasitism of fungus to cyanobacteria have limited the application of these two types of microorganisms [19]. Actinobacteria are distributed mainly in soil and produce several active substances, including antibiotics, enzymes, organic acids, amino acids, and peptides. In 1962, Safferman and Morris found that 90% of 213 actinobacteria strains exhibit algicidal activities [17]. Specifically, actinobacteria species such as *Streptomyces achromogenes*, *S.* exfoliatus, *S.* neyagawaensis, and *S. phaeofaciens* have been shown to exhibit algicidal abilities yet, similar to algicidal bacteria, few compounds have been purified and isolated [20–23].

In this study, an algicidal actinobacteria was isolated from the soil boarding a fresh water pond. This strain was identified as *Streptomyces* sp. L74 and exhibits algicidal activities that are harmful to cyanobacteria, *Microcystis aeruginosa*, *Anabaena flos-aquae*, *Oscillatoria animalis*, and *Aphanizomenon flos-aquae*. An algicidal compound from *Streptomyces* sp. L74 cultures was isolated, purified, and identified. The mechanism of algicidal activity of *Streptomyces* sp. L74 was also studied.

## Materials and Methods

### Ethics Statement

No specific permits were required for the described field studies in the center lake of Guanghzou Higher Education Mega Center (http://en.wikipedia.org/wiki/Guangzhou Higher Education Mega Center). The research sites are not privately-owned or protected in any way and field studies did not involve endangered or protected species.

### Isolation of Algicidal Actinobacteria

Soil samples were collected from the topsoil near the center lake of Guanghzou Higher Education Mega Center, where cyanobacterial blooms of *M. aeruginosa* usually form. The soil samples were air dried at room temperature, ground, and sieved. Soil powder (2 g) was suspended in phosphate buffer solution (PBS, pH 7.0) and diluted to 10^−2^, 10^−3^, 10^−4^, and 10^−5^. Approximately 0.1 mL of dilutions was spread on Gause’s synthetic agar medium plates [24]. Potassium dichromate (75 µg/L) was added in the medium as a growth inhibitor of actinobacteria as well as other bacteria and fungi [25]. The colonies were grown on plates at 28 °C for 7 d and those with different morphologies were selected and streaked onto new agar plates. The colonies were re-streaked several times to obtain purified isolates.

A modified double-layer agar plate method was used to isolate algicidal actinobacteria according to Yang et al. [26]. Double-layer agar plates contained 20 mL of basal agar BG-11 medium (2% agar) and over-layered soft agar medium. Soft-agar medium was made of 2 mL of cyanobacterial cell suspension at the exponential growth phase and 3 mL of BG-11 medium with 1% agar. After the cyanobacterial cells were cultivated in double-layer agar plates at 25 °C at a light intensity of 2000 lux for 5 d, Oxford cups containing the isolated actinobacteria colonies were placed on the surface of the agar plates. The double-layer agar plates were cultivated for another 5 d at 25 °C at a light intensity of 2000 lux. A clear zone around the Oxford cups on the double-layer agar plates indicated the algicidal activity of the isolate.

Positive strains were inoculated in fresh fluid of Gause’s synthetic medium and incubated for 2 d to determine the algicidal activity. Approximately 5 mL of the strain culture was added to 50 mL of cyanobacterial culture cultivated at 25 °C at a light intensity of 2000 lux and a flask shaking speed of 150 rpm. The concentration of cyanobacterial cells in the culture and the algicidal activity were determined based on optical density at 650 nm (OD_650_).

### Cyanobacterial Strains

Various cyanobacterial species such as *M. aeruginosa* FACHB 905, *A. flos-aquae* FACHB 245, *O. animalis* FACHB 943, and *Aph. flos-aquae* FACHB 943 were obtained from the FACHB collection (http://algae.ihb.ac.cn/), Institute of Hydrobiology, Chinese Academy of Sciences. The cyanobacterial strains were pre-cultivated in Erlenmeyer flasks at 25 °C, at a light intensity of 2000 lux (Intelligent Light Incubator GXZ-500D, Ningbo, China), and with light-dark cycles 14 h: 10 h to reach the exponential growth phase. BG-11 medium (http://microbiology.ucdavis.edu/meeks/BG11medium.html) was used to cultivate the strains.

### Morphology and 16S rRNA Gene Identification of Algicidal Actinobacteria Isolate

The morphology of the isolate was observed under a light microscope (Leica DM2500M, Germany). The spores were fixed and stained with 1% osmic acid, plated with gold film (BAL-TEC SCD-500, Germany), and observed by scanning electron microscopy (SEM, Philips XL-30, Holland).

Genomic DNA was extracted in accordance with the protocol of Takara Bio MiniBEST universal genomic DNA extraction kit ver. 4.0 (Takara Bio, Dalian, China). Primer pair 27F/1492R (27F: 5’-AGA GTT TGA TCC TGG CTC AG-3’; 1492R: 5’-TAC CTT GTT ACG ACT T-3’) was used for 16S rRNA gene amplification [27]. PCR amplification was prepared with 1.25 U of *Taq* DNA polymerase (Takara Bio, Dalian, China), 5 µL of 10× PCR buffer (15 mM Mg^2+^), 4 µL of dNTPs (2.5 mM), 4 µL of DNA template (50 ng), and 0.5 µL of each primer (20 µM) with a final volume of 50 µL. PCR amplification was ran for 30 cycles under the PCR conditions described previously [27]. The PCR products were examined by 1% (w/v) agarose gel electrophoresis in 1× Tris-acetate EDTA buffer and stained with ethidium bromide (0.5 µg·ml^−1^). The 16S rRNA gene was sequenced by Beijing Genomics Institute (Shenzhen, China).

The nucleotide-nucleotide BLAST (BLASTn) database (http://www.ncbi.nlm.nih.gov/BLAST), Seqmatch program, and CHIMERA_CHECK program of the Ribosomal Database Project (http://rdp.cme.msu.edu/) were used to analyze the 16S rRNA gene sequence of the isolates. The sequence and the closest matches retrieved from the database were aligned. Phylogenetic trees were constructed using the neighbor-joining method in MEGA 3 software [28].

### Determination of Chlorophyll a

Chlorophyll a (Chl *a*) content in the cyanobacterial culture was determined according to Wintermans et al. [29]. The cyanobacterial culture (V_1_) was centrifuged at 4,000 rpm for 10 min. The cell pellet was collected, resuspended in 95% (v/v) ethanol, and stored at 4 °C for 24 h. The supernatant (V_2_) was centrifuged at 4,000 rpm for 10 min and collected. A spectrophotometer (Unico UV-2802S, Shanghai, China) was used to determine light absorbance at 665 nm (Chl *a* absorption), 649 nm (Chl *b* absorption), and 750 nm (turbidity correction). Chl *a* concentration was determined based on Eq. (1):

$${\text{Chl a (mg/L)=[(A}}_{\text{665}}{\text{-A}}_{\text{750}}\text{)}\times \text{13}{\text{.7-(A}}_{\text{649}}{\text{-A}}_{\text{750}}\text{)}\times \text{5}\text{.76]}\times \frac{{\text{V}}_{\text{2}}}{\text{V1}}$$ (1)

### Extraction, Isolation, and Identification of Algicidal Compounds from *Streptomyces* sp. L74 Culture

*Streptomyces* sp. L74 was incubated by shaking in 250 mL of Gause’s synthetic liquid medium at 28 °C for 7 d. The bacterial culture was centrifuged at 5,000 rpm for 10 min. The culture supernatant was recovered by filtering through a 0.22 µm microporous membrane. A fourfold volume of ethanol was added slowly to the culture supernatant; the solution was mixed for 30 min at room temperature and stored at 4 °C for 2 h. The supernatant was centrifuged at 5,000 rpm for 10 min and collected. After ethanol evaporated in a rotary vacuum evaporator (Rikakikai NE1001, Japan) at 40 °C, the supernatant was extracted continually thrice by using an organic solvent at equal volume. After the solvent evaporated under reduced pressure at 40 °C, the residue was fractionated by silica-gel flash column chromatography (Bio-Rad DuoFlow, America) and eluted with chloroform in CH_3_OH (15:4, v/v). The fraction exhibiting algicidal activity was subjected to size-gel exclusion chromatography (16 mm ID × 40 cm, Sephadex G15) by using ultrapure water as an eluent. An active fraction was placed in a reversed-phase C_18_ column (250 mm × 4.6 mm, Agilent) connected to a high-performance liquid chromatography (HPLC) system (Waters 1525, Waters) and monitored at 210 nm. Elution was performed with 90% (v/v) CH_3_OH aqueous solution at a flow rate of 0.5 mL/min for 20 min. The algicidal activities of the fractionated compounds were identified by determining the concentration of Chl *a* in *M. aeruginosa* in BG-11 medium.

The active algicidal compound was dried in vacuum (Christ Alphal-2, Germany) and scanned by SEM to identify the morphology of the compound. The active algicidal compound was filled with potassium bromide at 20 MPa, and the infrared spectrum (IR) of the active algicidal compound was analyzed in Vector 33 (Bruker, Germany). Electrospray ionization-mass spectrum (ESI-MS) was obtained using a Waters 1525 HPLC coupled with an Esquire HCT (Bruker, Germany). The purified active algicidal compound was dissolved in deuterium oxide (CIL, America) and placed in a nuclear magnetic resonance (NMR) spectrometer (Bruker AV400D, Germany) operating at 400 MHz for ^1^H NMR and 100 MHz for ^13^C NMR.

### Determination of Superoxide Dismutase, Catalase, and Peroxidase Activities as well as Malonaldehyde in *M. aeruginosa*

Approximately 1 mL of compound B3 water solution from HPLC was added to 99 mL of *M. aeruginosa* culture at the exponential growth phase. The cyanobacterial cells from different co-culture times were collected by centrifugation at 8,000 rpm and 4 °C for 10 min. The cyanobacterial cells were suspended with phosphate buffer saline (PBS, pH 7.8) and ultrasonicated at 400 W for 5 min. The supernatant was collected by centrifugation at 10,000 rpm and 4 °C for 20 min and then stored at -70 °C. This process was performed until enzyme activity was determined. Superoxide dismutase (SOD) activity was determined by nitro blue tetrazolium photoreduction method [30]. Catalase (CAT) activity was determined by ultraviolet spectrophotometry [31]. Peroxidase (POD) activity was determined by Guaiacol method [32]. Malondialdehyde (MDA) was determined using the thiobarbituric acid method [33].

### Nucleotide Sequence Accession Number

The sequence reported in this study was deposited in the GenBank database with accession number JX983654.

## Results

### Screening and Identification of Algicidal Actinobacteria

**Figure 1:**
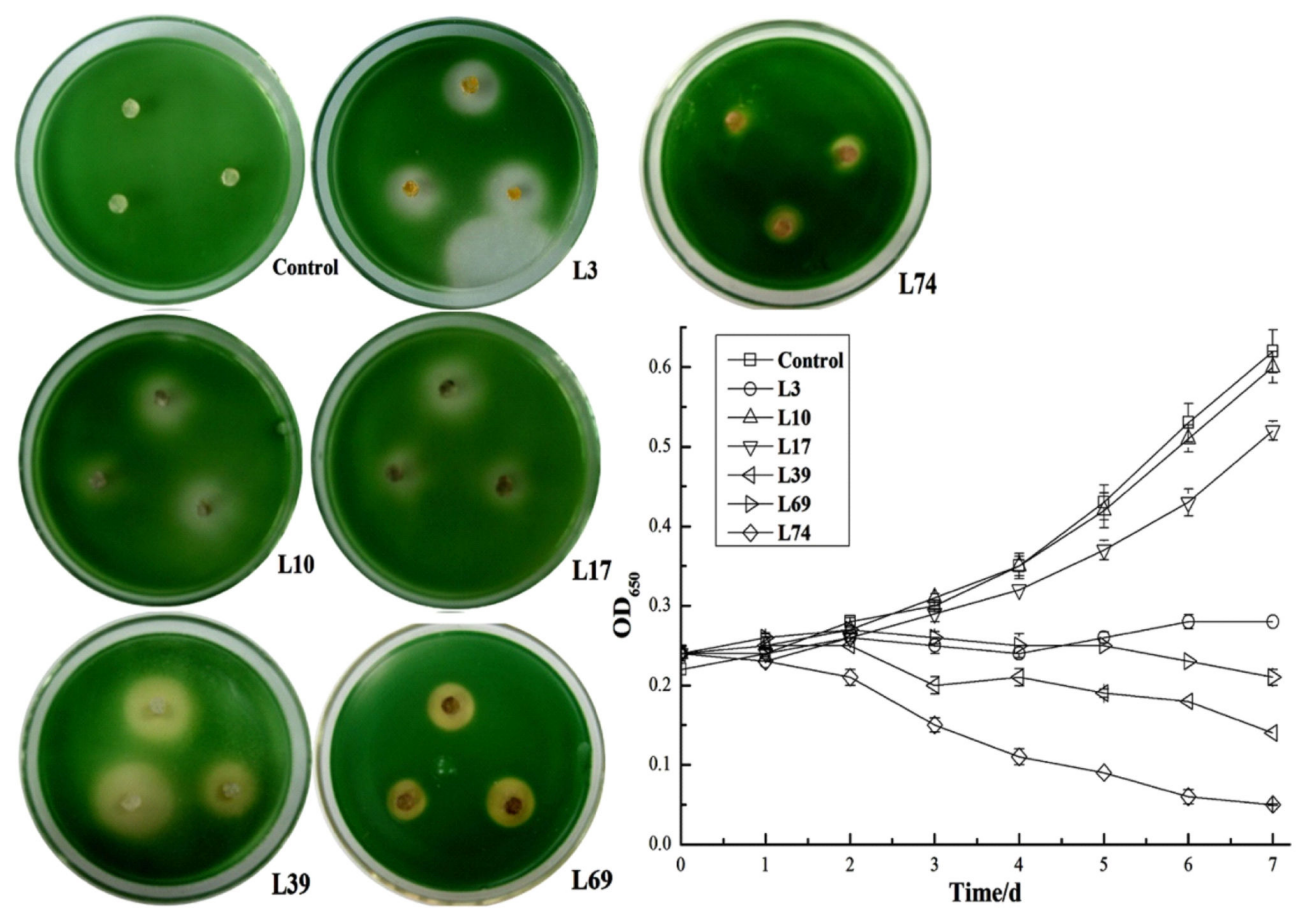
Algicidal activity of actinobacteria isolates on *M. aeruginosa* agar plates and fluid *M. aeruginosa* cultures.

A total of 117 actinobacteria isolates were obtained from the soil. Among these isolates, six isolates, namely, L3, L10, L17, L39, L69, and L74, exhibited algicidal activity against *M. aeruginosa*. Isolate L39 in double-layer agar plate exhibited the largest algicidal zone. In the fluid containing *M. aeruginosa*, isolate L74 exhibited the highest algicidal activity (Figure 1). After repeat screening, isolate L74 was chosen as the targeted algicidal strain and applied for the next experiments.

**Figure 2:**
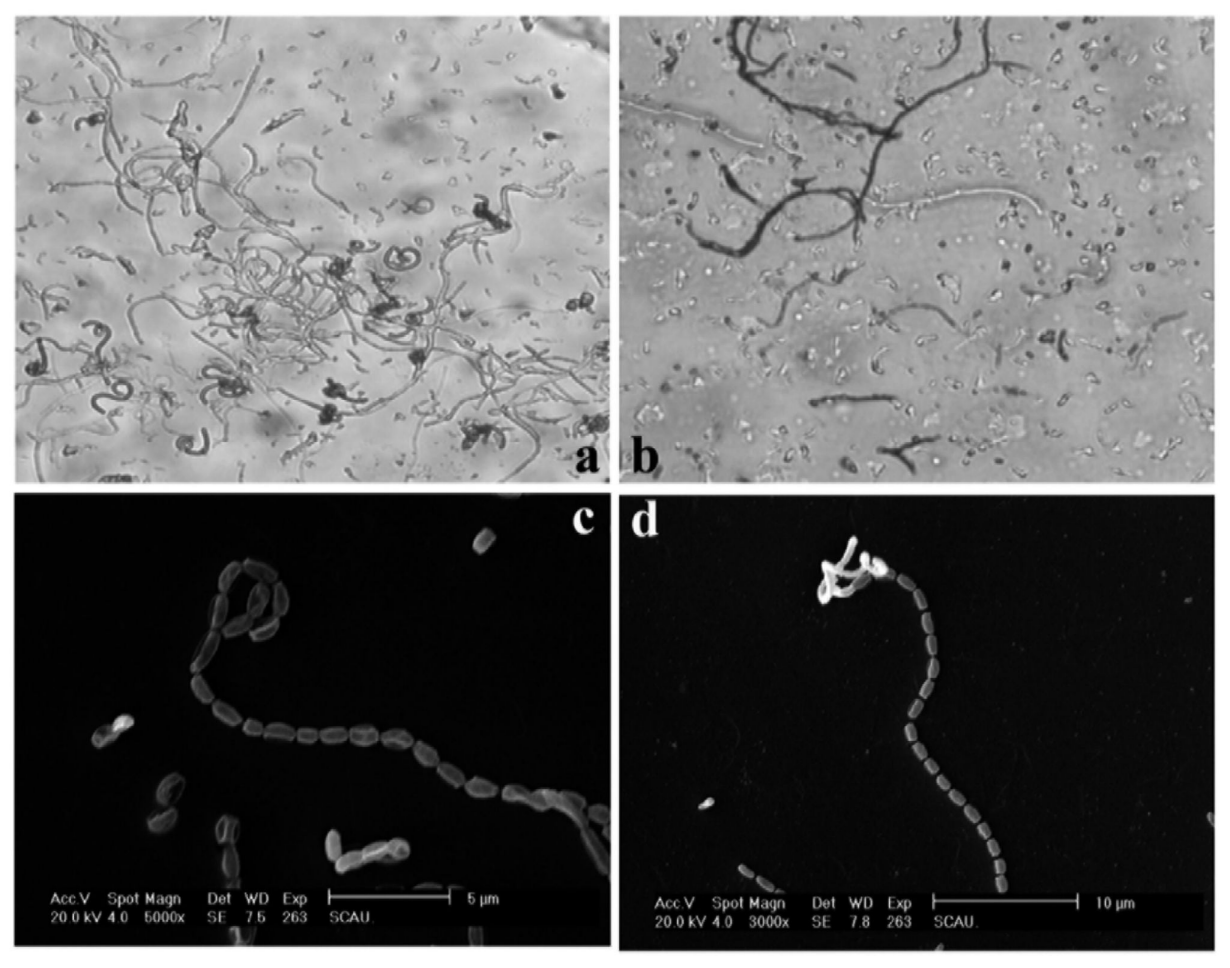
Hyphae and spores of strain L74. a and b: light microscope ×500; c: SEM ×6,000; d: SEM ×3,000.

Strain L74 exhibited the following morphological characteristics on the agar plate: substrate; aerial; and spore hyphae. The spore hyphae were faint pink straight or winding chains under a light microscope (Figure 2a and 2b). The spores were oval, approximately 1 µm × 0.5 µm to 1 µm in size, and grew separately (Figure 2c and 2d). The microscopic results suggested that strain L74 possibly belongs to the genus *Streptomyces*.

**Figure 3:**
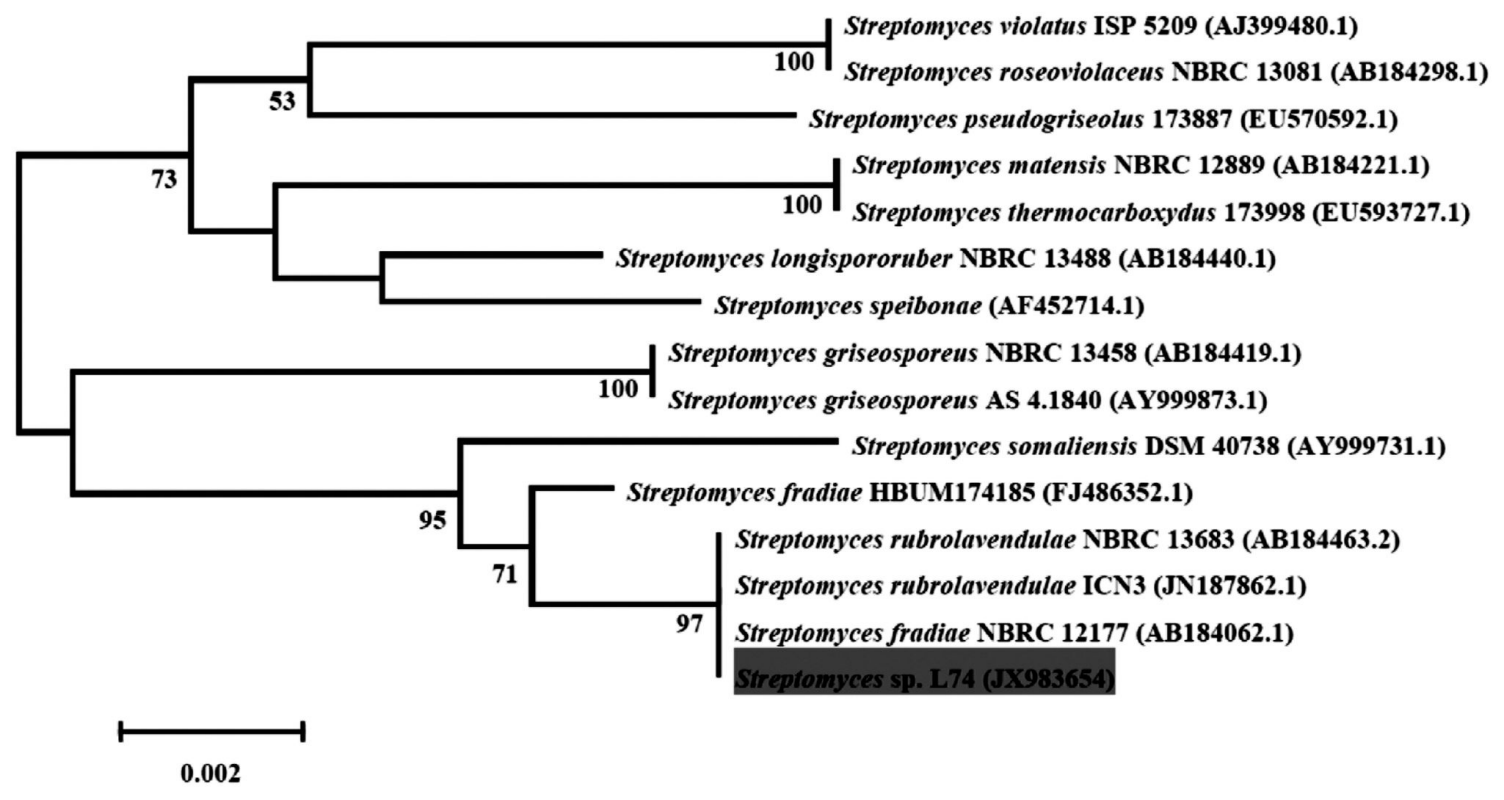
Phylogenetic tree based on the 16S rRNA gene of strain L74. The tree was inferred using the neighbor-joining method, and evolutionary distances were computed using the Kimura’s 2-parameter model. Bootstrap values (1,000 replicates) above 50 are shown at each node.

After sequencing, the 16S rRNA gene of strain L74 (comprising 1393 bp nucleotides) was determined. BLASTn and RDP databases revealed that the sequence of strain L74 is closely related to the genus *Streptomyces* and exhibited 100% sequence similarity to *S. fradiae* or *S. rubrolavendulae*. Considering strain similarity and phylogenetic analysis, we found that strain L74 was *Streptomyces* sp. L74. A phylogenetic tree was constructed using the 16S rRNA gene of the strain and its closely related sequences in the NCBI database (Figure 3).

### Algicidal Characteristics of *Streptomyces* sp. L74

**Figure 4:**
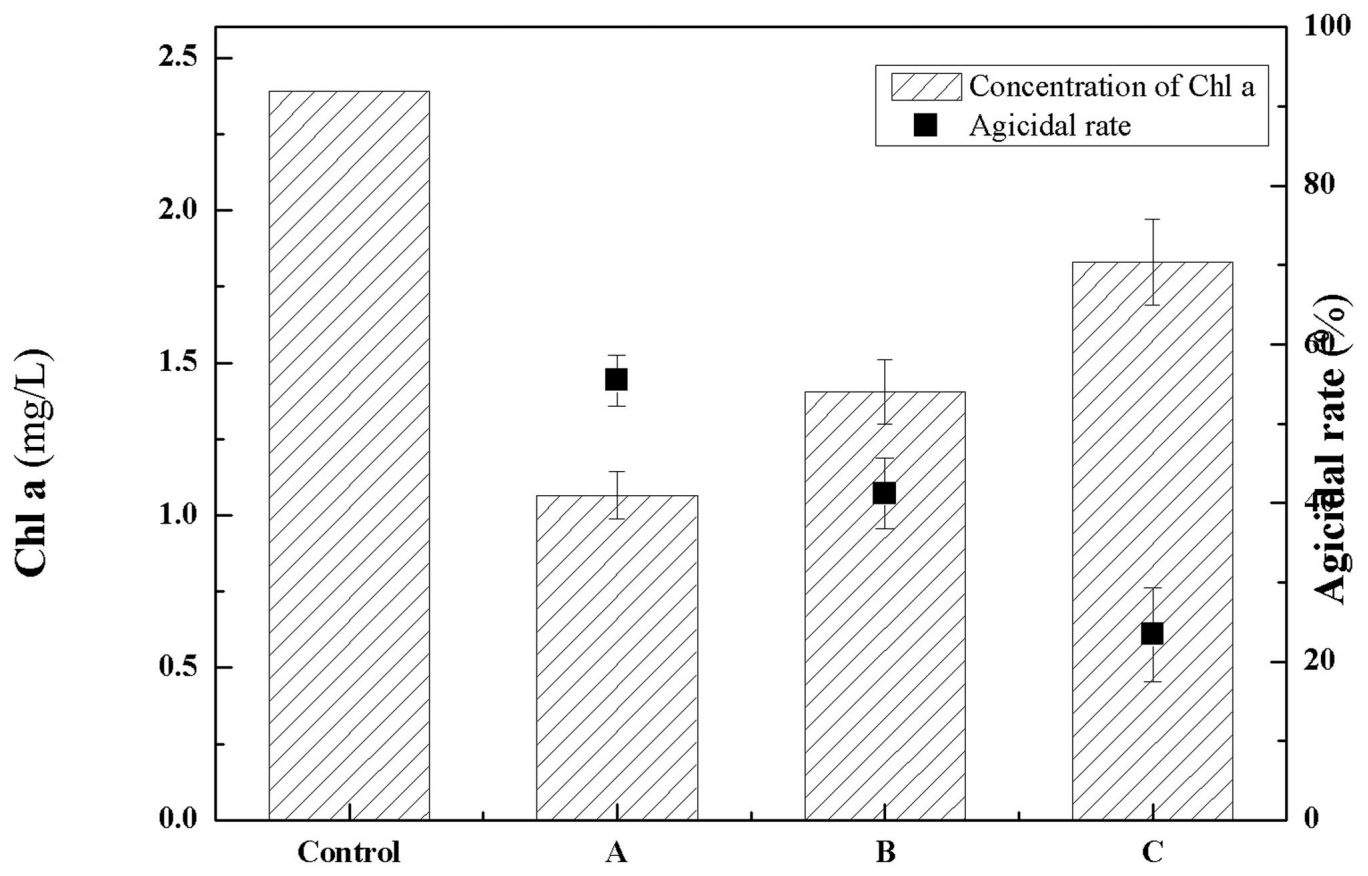
Algicidal effects of *Streptomyces* sp. L74 cultures to *M. aeruginosa*. Control: *M. aeruginosa* cultures without addition of *Streptomyces* sp. L74 cultures; A: *M. aeruginosa* cultures with addition of *Streptomyces* sp. L74 cultures; B: *M. aeruginosa* cultures with addition of *Streptomyces* sp. L74 cell-free cultures after filtration with 0.22 µm microposous membrane; C: *M. aeruginosa* cultures with addition of *Streptomyces* sp. L74 cells after centrifugation and washing with distilled water.

The Chl *a* concentration of cyanobacterial cultures indicated that the growth of *M. aeruginosa* was affected by *Streptomyces* sp. L74 cultures with an algicidal rate of 55.23%, while *Streptomyces* sp. L74 cell-free cultures and washed *Streptomyces* sp. L74 cells exhibited algicidal rates of 41% and 23.4% to *M. aeruginosa*, respectively (Figure 4). These results suggested that strain L74 exhibits an algicidal activity mainly by an indirect attack of secreted active compounds and partially by a direct attack of bacterial cells.

Strain L74 exhibited an algicidal activity against *M. aeruginosa, A. flos-aquae*, *O. animalis*, and *Aph. flos-aquae* (Table 1). Strain L74 showed a more rapid algicidal effect on *M. aeruginosa* at 2 d when the algicidal rate was three times to six times higher than the other tested cyanobacteria. The algicidal activities against *M. aeruginosa*, *O. animalis*, and *Aph. flos-aquae* are mainly facilitated by an indirect attack of algicidal compounds of strain L74 (80% of filter liquor in the cultures). By contrast, algicidal activity against *A. flos-aquae* is mainly facilitated by a direct attack of strain L74 cells (40% of filter liquor in the cultures).

**Table 1:** Algicidal effects of *Streptomyces* sp. L74 cultures to testing cyanobacteria.

Strains	Algicidal rate^a^ by cultures (%)				Algicidal rate by filter liquid^b^ (%)		
	2 d	4 d	6 d		2 d	4 d	6 d
*M. aeruginosa* FACHB 905	57.78±7.61	71.48±5.33	82.59±5.67		40.33±5.85	53.00±1.00	62.67±4.04
*A. flos-aquae* FACHB 245	9.62±1.15	43.20±5.58	85.64±0.80		18.80±4.04	36.15±1.54	37.56±1.35
*O. animalis* FACHB 943	13.33±12.71	32.59±13.11	75.56±1.71		7.41±6.41	28.89±5.56	62.96±0.43
*Aph. flos-aquae* FACHB 943	17.92±5.67	46.88±14.38	71.88±3.48		6.46±9.38	25.00±9.01	57.50±6.38

a determined by the concentration of Chl a after adding 5 mL of *Streptomyces* sp. L74 cultures with initial cells concentration of 1×10^6^ to 50 mL aglae culture; b: filtrated with 0.22 µm microposous membrane.

### Physical and Chemical Characteristics of Algicidal Compounds

**Figure 5:**
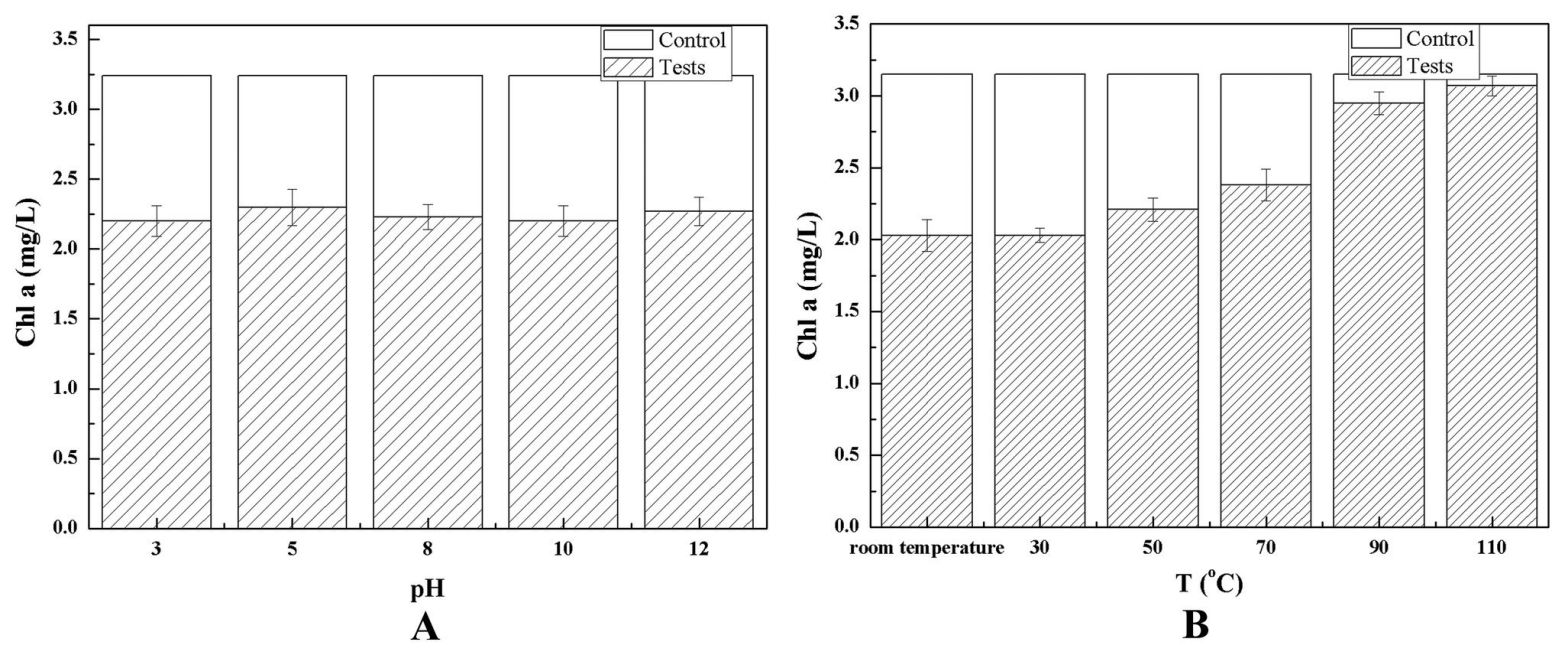
Effects of pHs (A) and temperatures (B) on algicidal activity of algicidal compounds to *M. aeruginosa*.

The algicidal compounds considered as hydrophilic substances were extracted by mineral ether, acetic ether, or butyl alcohol and subsequently dissolved in the water phase. The algicidal compounds were dialyzed out of a 3 kDa bag filter, indicating that these substances have molecular weights of <3 kDa. The algicidal compounds were stable at 70 °C with pH ranging from 3 to 12 (Figure 5).

The stability of the algicidal compounds to heat and pH indicated that these compounds were not proteins or peptides. Table 2 shows that the specific chemical color reactions of algicidal compounds with reducing sugars, polysaccharides, glycosides, and triterpenes were positive. These results suggested that the algicidal compounds may contain radical groups of reducing sugars, polysaccharides, glycosides, and parent nuclei of triterpenes.

**Table 2:** Chemical identification of algicidal compounds.

**Testing items**	**Methods**	**Results**
Alkaloids	Mercury potassium iodide test	-
Amino acids, peptides or proteins	Ninhydrin test	-
Reducing sugars, Polysaccharides or glycosides	α-naphthol test	+
Organic acids	Bromophenol blue test	-
Phenols	Ferric chloride test	-
Triterpenes	Glacial acetic acid - sulfuric acid test	+
Steroids	Glacial acetic acid - sulfuric acid test	-
Steroids	Chloroform - sulfuric acid test	-
Cardiac glycosides	Alkaline picric acid test	-
Anthraquinones	Boric acid test	-
Flavonoids or flavonoid glycosides	Aluminum chloride test	-
Lactone or lactone glycosides	Hydroxamic acid iron test	-

negative; + positive.

### HPLC Purification and Identification of Algicidal Compounds

**Figure 6:**
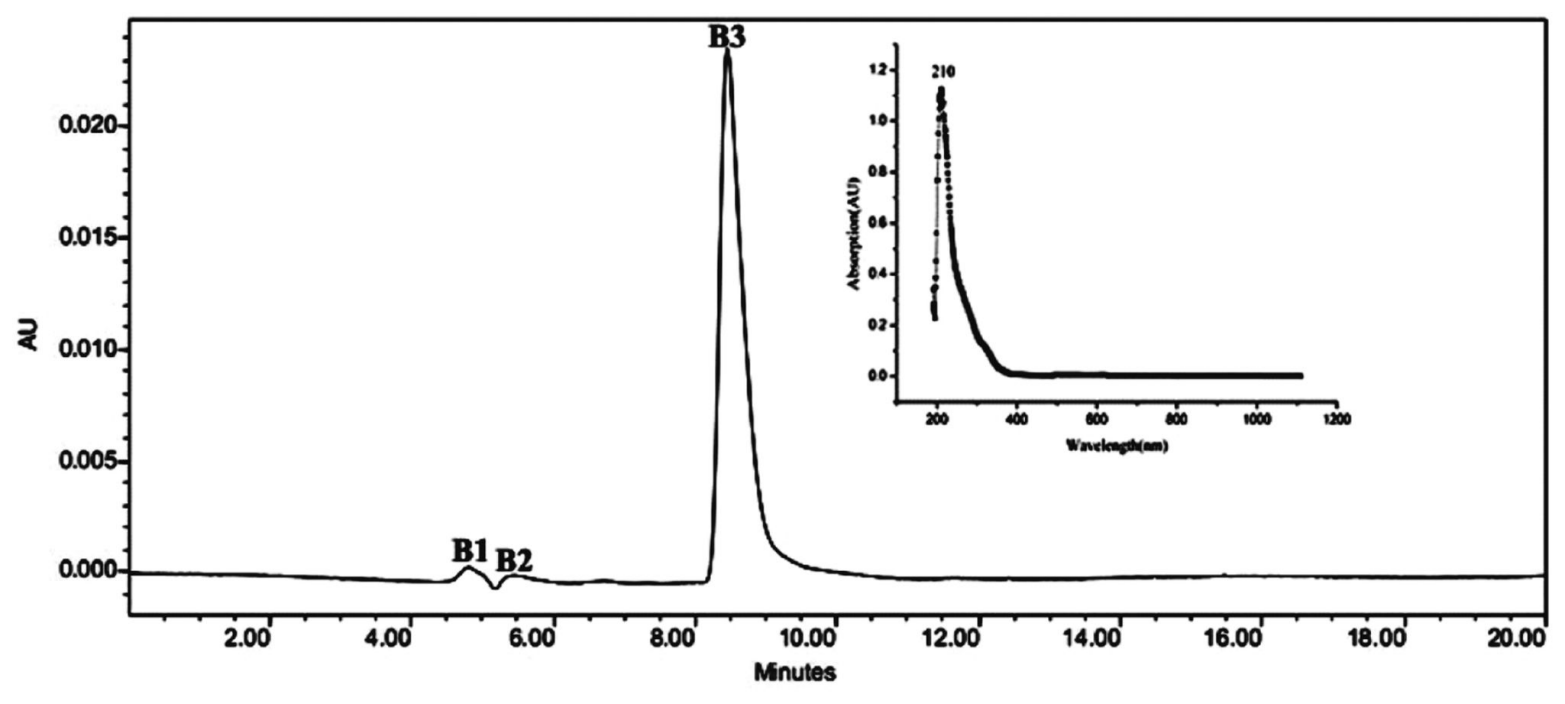
HPLC and UV absorption spectrum of active fraction from Sephadex G15 column.

HPLC results suggested that the three compounds B1, B2, and B3, which eluted at the following retention times: 4.5 min to 5 min, 5 min to 6 min, and 8 min to 10 min, respectively, were present in the active fraction separated by size-gel exclusion chromatography (Figure 6). B3 compound exhibited an algicidal activity against *M. aeruginosa* and was applied for the next identification of algicidal mechanism.

**Figure 7:**
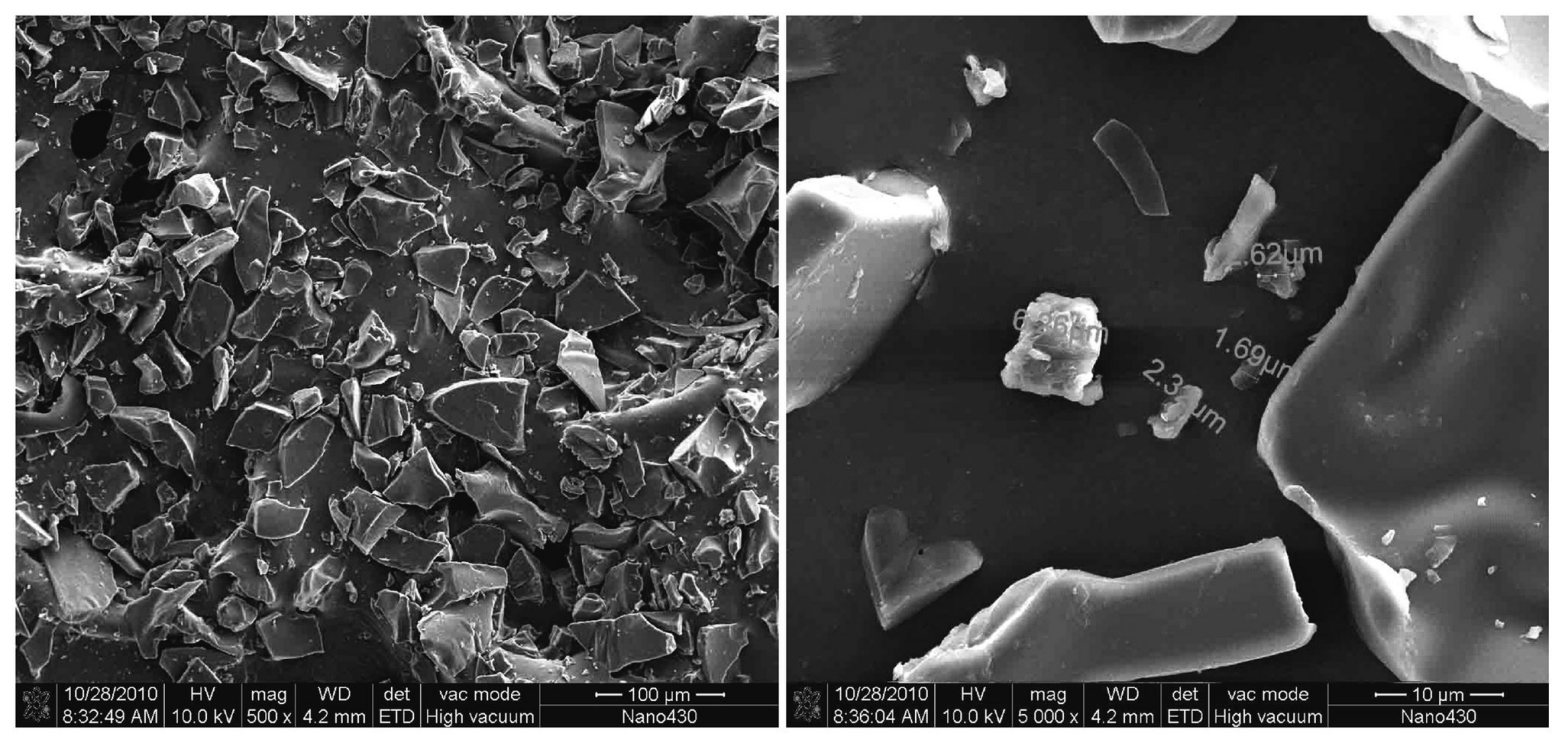
Morphology of algicidal compound B3 in SEM.

**Figure 8:**
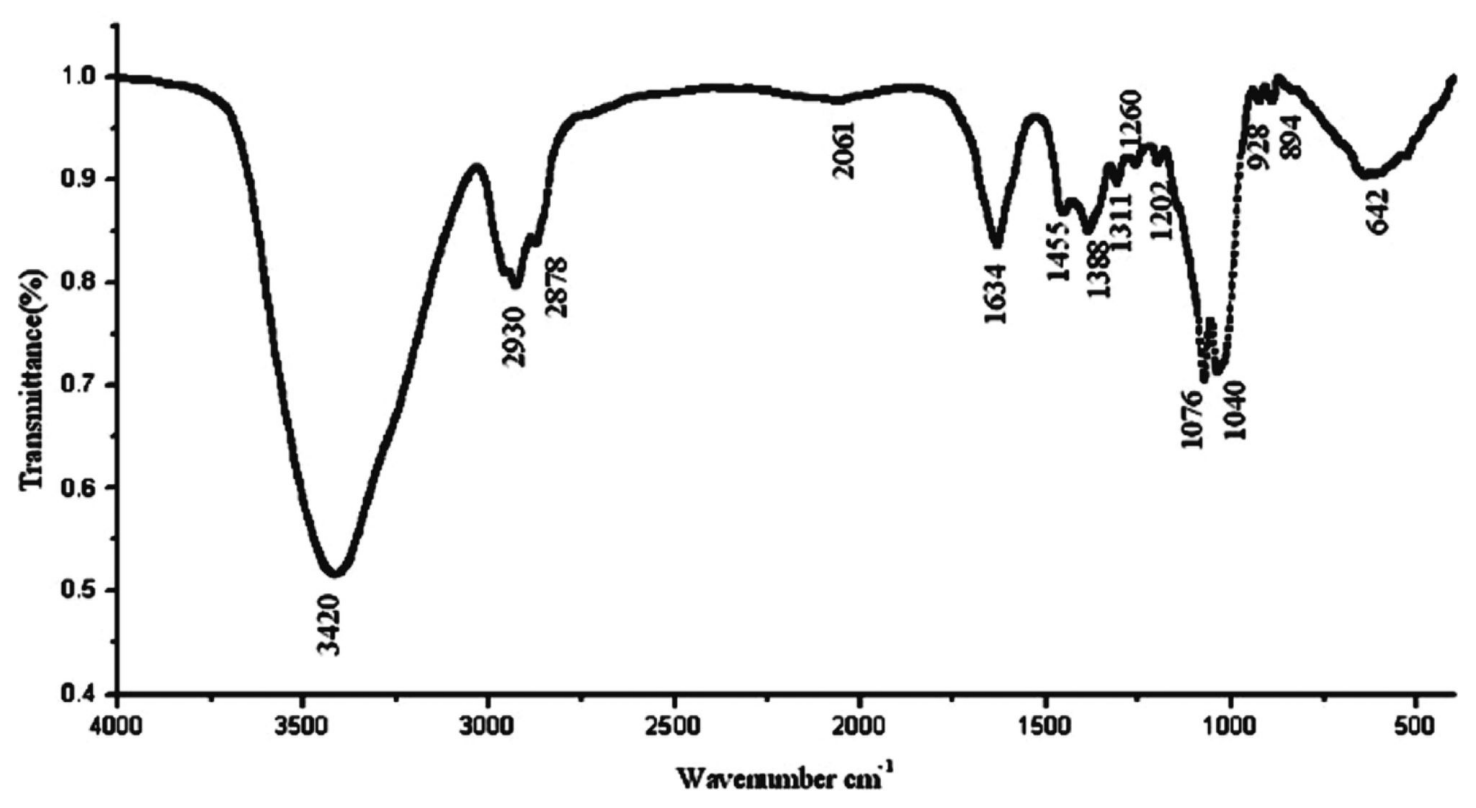
Infrared spectroscopy of algicidal compound B3 packed into potassium bromide.

SEM revealed that the morphology of algicidal B3 compound exhibited indefinite forms. B3 compound was successfully obtained at high purity (Figure 7). Figure 8 shows that the IR absorption spectra of the residual groups of B3 were at 3420, 2930, 2878, 1634, 1455, 1388, 1311, 1260, 1202, 1076, 1040, 928, and 894 cm^−1^. The IR absorption at 3420 cm^−1^ indicated the presence of several –OH groups. In addition, a larger peak area corresponds to a higher number of –OH groups. The IR absorption at 1634 cm^−1^ indicated the presence of unsaturated -C=C-. IR absorption at 1040 cm^−1^ indicates the presence of glycosidic bonds. The active algicidal B3 compound could be a type of saponin based on IR spectrum analysis and comparison with saponins in the database. ESI-MS was recorded in a positive mode and yielded a deprotonated molecule at *m*/*z* 782.7 [M+H]^+^, *m*/*z* 602.7 [M+ H -glc]^+^, and *m*/*z* 422.7 [M+ H -2glc]^+^. [M+H]^+^ ion showed that the molecular mass of B3 compound could be determined at *m*/*z* 782.7. The molecular distinction between *m*/*z* 782.7 and 602.7 and between *m*/*z* 602.7 and 422.7 was 180. These distinctions are consistent with the IR result, indicating the presence of glycosidic groups in B3 compound. Two glucosyl groups are also present in the B3 compound structure. Similarly, the specific chemical color reactions and IR spectrum analysis revealed a nucleus of terpenoid with a molecular weight of 422.7 in the B3 compound structure. NMR spectral data analysis showed that the molecular formula of B3 compound is C_42_H_70_O_13_.

**Figure 9:**
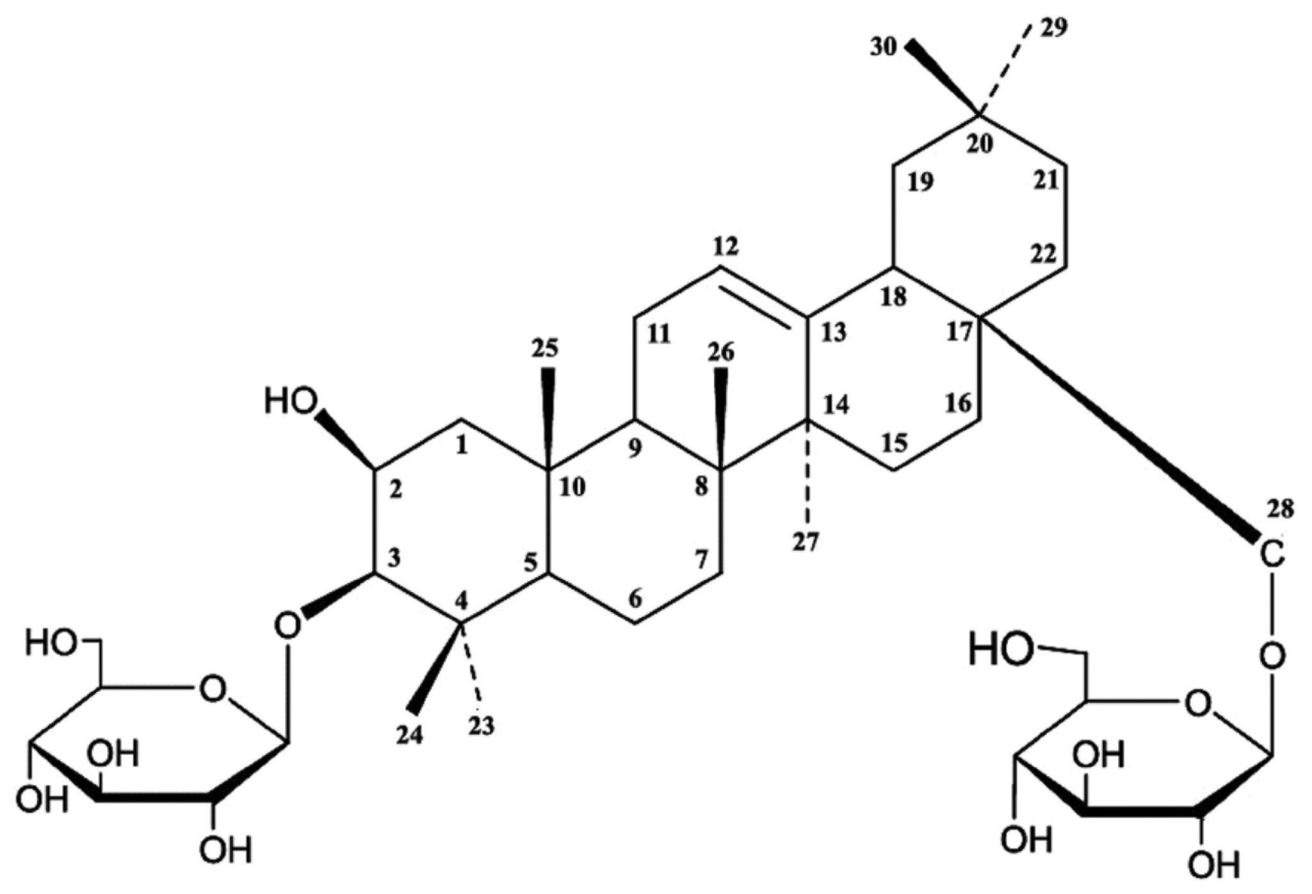
Structure of compound B3.

^13^C NMR spectrum data indicated the presence of two alkenyl carbons (124.6 and 132.8 ppm, C12 and C13, respectively) and at least 12 C–O carbons (40 ppm to 90 ppm) in the B3 compound (Table 3). IR spectrum and ESI-MS analyses suggested that one ethylenic bond and two glucosyl groups are present in the compound. The absence of carbonyl groups (150 ppm to 200 ppm) in the ^13^C NMR spectrum data suggested that keto-, carboxy-, and ester groups were present in the molecular structure of B3. The ^1^H NMR spectrum further suggested that seven methyl groups (C23, C24, C25, C26, C27, C29, and C30) and one alkenyl group (5.15 ppm, C12) were present. These results are consistent with IR results. Among the 42 carbons, 12 and 30 were assigned to the sugar units comprising 2 hexoses and the triterpene moieties, respectively. The triterpene moieties were correlated with those in previous studies. The δ values of C3 at 84.5 ppm and C28 at 69.6 ppm as well as the ESI-MS spectrum (two glucosyls with *m*/*z* 180) suggested that the triterpenoid saponin is a bisdesmosidic glycoside with a hexose attached to C3 and C28 positions. These data showed that the structure of compound B3 is 2-hydroxy-12-oleanene-3,28-*O*-D-glucopyranosyl (Figure 9).

**Table 3:** ^1^H NMR (D_2_O, 400 MHz) and ^13^C NMR (D_2_O, 100 MHz) spectral data for algicidal activie compound B3.

**Position**	**δ ^13^C (ppm)**	**δ ^1^H [m, *J* (Hz)]**
1	38.2	1.09
2	60.9	4.12
3	84.5	4.07
4	38.7	-
5	59.6	0.92
6	17.4	1.17
7	30.2	1.20
8	40.4	-
9	47.7	1.49
10	35.2	-
11	22.9	1.97
12	124.6	5.15
13	132.8	-
14	48.7	-
15	30.1	1.19
16	25.9	1.99
17	51.2	-
18	43.7	3.22
19	51.6	2.21
20	29.3	-
21	25.2	1.11
22	16.2	2.05
23	60.8	1.33 (s)
24	25.8	1.24 (s)
25	15.1	1.06 (s)
26	16.8	1.63 (s)
27	16.7	1.70 (s)
28	69.6	4.46
29	39.0	0.94 (s)
30	22.0	0.95 (s)
C28-O-glc		
1’	96.3	4.61 (d, 7.4)
2’	73.6	3.14
3’	79.0	3.38
4’	71.0	3.29
5’	76.8	3.41
6’	69.4	3.69, 3.81
C3-O-glc		
1’’	104.1	4.70 (d, 7.4)
2’’	75.7	3.12
3’’	76.1	3.36
4’’	73.5	3.27
5’’	81.1	3.44
6’’	61.1	3.68, 3.78

### Algicidal Mechanism of B3 Compound Secreted by *Streptomyces* sp. L74

**Figure 10:**
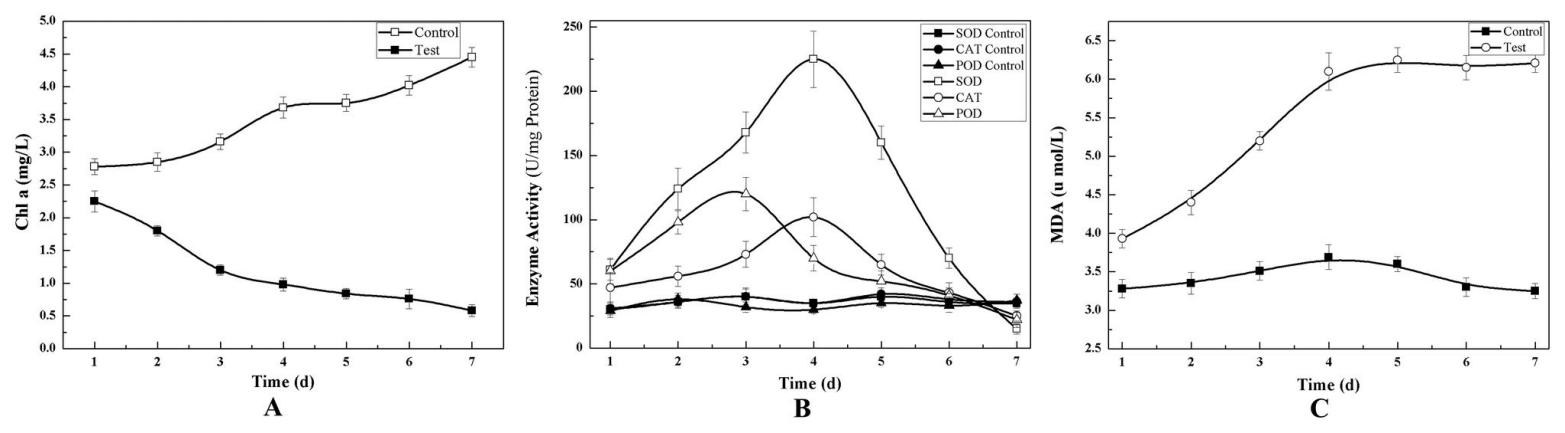
Effects of algicidal compound B3 to *M. aeruginosa*. Growth (A), Superoxide Dismutase (SOD), Catalase (CAT), and Peroxidase (POD) Activity (B) and Malonaldehyde (MDA) concentration (C).

The purified compound B3 exhibited an activity against *M. aeruginosa* (Figure 10 A). The concentration of cyanobacterial cells decreased from 2.25 mg/L to 0.58 mg/L after these cells were co-cultured with the active compound for 7 d. By contrast, the concentration of cyanobacterial cells increased from 2.78 mg/L to 4.45 mg/L in the non-B3 cyanobacterial culture (Figure 10 A). The SOD, CAT, and POD activities of *M. aeruginosa* in the co-culture with compound B3 were affected by the active compound when SOD activity increased from 60.95 U/mg·protein to 225.83 U/mg·protein after 4 d. The SOD activity of the compound decreased to 19.99 U/mg·protein at 7 d. CAT activity increased from 46.96 U/mg·protein to 101.90 U/mg·protein at 4 d and then significantly decreased to 25.79 U/mg·protein at 7 d. POD activity increased from 59.56 U/mg·protein to 120.78 U/mg·protein at 3 d and then decreased greatly to 22.11 U/mg·protein at 7 d (Figure 10 B). After the cells were co-cultured with B3 compound, the MDA concentration of *M. aeruginosa* increased from 3.93 µmol/L to 6.21 µmol/L at 5 d. This concentration was maintained for the next cultural time (Figure 10 C). The antioxidant systems of the cyanobacterial cells were activated immediately after such cells reacted with B3 compound. These cells were damaged after they reached their limits. MDA is a product of lipid oxidation. The MDA concentration in *M. aeruginosa* indicates the oxidation status of the lipid membrane, maximum MDA limit, and severity of cell damage. *Streptomyces* sp. L74 released the active compound, thereby exhibiting an algicidal activity against *M. aeruginosa* by destroying the antioxidant systems of the cyanobacterial cells.

## Discussion

*S. achromogenes*, *S.* exfoliatus, *S.* neyagawaensis, and *S. phaeofaciens* are actinobacterial species exhibiting algicidal abilities [20–22]. This study is the first to report about an actinobacterial strain closely related to *S. fradiae* and *S. rubrolavendulae* with algicidal abilities. This study is also the first to identify an algicidal compound as a type of a triterpenoid saponin from the strain culture. The algicidal mechanism of this active algicidal compound was also determined based on the toxicity to antioxidant systems of cyanobacterial cells.

*Streptomyces* sp. L74 exhibited algicidal activity against *M. aeruginosa*, *A. flos-aquae*, *O. animalis*, and *Aph. flos-aquae*. *Streptomyces* sp. L74 indirectly attacks *M. aeruginosa*, *O. animalis*, and *Aph. flos-aquae* by using the active substances. *Streptomyces* sp. L74 directly attacks *A. flos-aquae* by cell-to-cell contact. *Cytophaga*, *Myxobacter*, and *Saprospira* from aquatic systems exhibit algicidal abilities by direct attack; by contrast, *Bacillus*, *Pseudomonas, Alteromonas*, and *Pseudoalteromonas* exhibit algicidal activities by indirect attack [14]. *Streptomyces* sp. L74 shows two patterns of algicidal activities. This species also exhibits high algicidal activities against harmful types of cyanobacteria. Therefore, this study provids basic information for a wide application of cynaoHAB control in various environments.

The active algicidal compound isolated from *Streptomyces* sp. L74 cultures was identified as a triterpenoid saponin with a molecular weight of 782. Several heat tolerant and pH stable algicidal compounds with molecular weights of <2 kDa have a wide range of algicidal activities. However, the active component in these compounds remains unidentified [13,23]. Yamamoto et al. [22] isolated L-lysine from the algicidal actinobacteria *S.* phaeofaciens culture and identified this microorganism as one of the causes of *M. aeruginosa* lysis. Triterpenoid saponin is a type of saponin found in many plants. These compounds exhibit antimicrobial, antibacterial, antifungal, antiviral, antiyeast, and cytotoxic activities [34–37]. Triterpenoid saponins are seldom produced by microorganisms. Shigematsu et al. [38] reported a tetracyclic triterpene glucoside extracted from the fermentation broth of fungus F11605. Pelaez et al. [39] extracted a triterpene glucoside named “enfumafungin” from *Hormonema* strain closely related to *Kabatina*. The compound exhibits antifungal activity against *Candida* and *Aspergillus*. Several scientists in China detected and extracted steroidal saponins and diosgenin from endophytic fungi and actinobacteria isolated from *Paris polyphylla* var. *Chinensis*. Several bacteria can convert ginsenoside to saponins [40,41]. Diterpene glucosides are parts of terpene glucoside compounds and produced by a fungus [42–44]. In contrast to the structures of known triterpenoid saponins, triterpenoid saponin found in this study may be a new type of saponin from actinobacteria.

Algicidal microorganisms kill cyanobacteria by indirect attack, in addition to direct cell-to-cell attack, via proteins, peptides, amino acids, antibiotics, nitrogen compounds, alkaloids, and other materials [8–14]. Various agents produced by algicidal microorganisms exhibit algicidal activities. However, few algicidal mechanisms have been determined. For instance, Yamamoto et al. [22] revealed that L-lysine from *S. phaeofaciens* culture exhibits algicidal activity by destroying the cyanobacterial cell wall. Lee et al. [8] identified a serine protease from the supernatant of *Pseudoalteromonas* sp. and suggested that the algicidal effect occurs via the enzymolysis of cyanobacterial protein by this protease. In the current study, the algicidal activity of *Streptomyces* sp. L74 against *M. aeruginosa* is caused by the destruction of the antioxidant systems of the cyanobacterial cells by the active terpenoid saponin. SOD, CAT, and POD are three important antioxidases in cells that protect organisms against damages caused by oxygen-free radicals. In 1969, Mccord and Fridovich [45] proposed a theory of superoxide radical damage to organisms. This theory has been widely applied in toxicology.

In conclusion, the algicidal activity of *Streptomyces* sp. L74 against harmful bloom-forming cyanobacteria is achieved by an indirect attack. Algicidal compounds are types of hydrophilic substances that are heat tolerant and pH stable. The purified active algicidal B3 compound was identified as a type of triterpenoid saponin (2-hydroxy-12-oleanene-3, 28-O-D-glucopyranosyl) with a molecular formula of C_42_H_70_O_13_. The algicidal mechanism of *Streptomyces* sp. L74 against *M. aeruginosa* is suggested to occur by disrupting the antioxidant systems of cyanobacterial cells by the active algicidal compound. Active algicidal compounds should be isolated and identified from *Streptomyces* sp. L74 to promote the application of this species in the control and treatment of cyanoHABs in various environments. The algicidal mechanism of these active algicidal compounds should also be further investigated. Further studies should be conducted to confirm the structure of the active algicidal B3 compound. The toxicity of this compound to aquatic organisms and persistence in the environment should also be investigated.
